# A simplified economic model for inertial fusion

**DOI:** 10.1098/rsta.2020.0053

**Published:** 2020-10-12

**Authors:** Nicholas Hawker

**Affiliations:** First Light Fusion Ltd., Unit 10 Oxford Industrial Park, Mead Road, Yarnton, Oxfordshire, OX5 1QU

**Keywords:** fusion, inertial fusion, LCOE, gain

## Abstract

A simple model for the levelized cost of electricity (LCOE) of an inertial fusion power plant is developed. The model has 14 parameters. These have been designed to be technology agnostic, such that the model may be applied broadly to all variants of inertial fusion. It is also designed to allow easy use of proxies from existing technology. The variables related most intimately to the physics challenges of inertial fusion, such as gain and target cost, are treated as parameters such that requirements can be found without bringing complex physics into the model. A Monte Carlo approach is taken to explore the parameter space. The most important conclusion is that a combination of high gain (greater than 500) and high fusion energy yield per shot (greater than 5 GJ) together appear to unlock more cost competitive designs than those in the existing literature. Designs with LCOE as low as $25/MWh are found with optimistic but not obviously unrealistic inputs.

This article is part of a discussion meeting issue ‘Prospects for high gain inertial fusion energy (part 1)'.

## 1. Introduction

Cost effective energy production using inertial fusion depends on many factors but one of the most important is the concept of ‘gain'. This can be defined in many ways but it is common in the inertial fusion community to talk in terms of ‘target gain', which is the fusion energy released divided by the energy input to the target. In these terms, it is often stated that a gain of 30–100 is required for power production from inertial fusion [1]. This conclusion comes from consideration of the driver and thermodynamic efficiencies, and a requirement for the recirculating power to be a small fraction of the total electrical power. Taken literally, the conclusion on required gain is true; a gain in that range is necessary for power production, but the analysis says nothing about cost. Fusion must be cost competitive in a market of different energy technologies.

### (a) The cost of electricity

Cost comparisons of energy generation technologies are typically made using the levelized cost of electricity (LCOE), which will be defined in equation (2.1). To understand the cost requirements for fusion means projecting the expected costs of future energy generation technologies to the time where a fusion power plant is expected to be built. Projections of this kind are not a precise science and are subject to substantial judgement, but with that caveat there are several major studies to draw on [2–4]. A summary of rough predicted values for the cost of electricity in the 2030s is given in table 1. These are indicative global averages; the actual cost depends strongly on the local economy and geography, particularly for renewables.

**Table 1.** A comparison of indicative costs of electrical generation for different technologies in the 2030s. Also shown is a whole-system cost for managing the intermittency of renewable generation, and a cost for large-scale carbon capture and storage (CCS).

	LCOE ($/MWh)	flexibility cost ($/MWh)	CCS cost ($/MWh)
solar PV	35	+15	—
onshore wind	35	+15	—
offshore wind	50	+15	—
coal	50	—	+70
gas	80	—	+70
nuclear	100	—	—

Renewables are intermittent by nature and to achieve a very high fraction of electricity generation requires a balancing of variability in the system. One estimate of the additional cost, from a whole-system point of view, of balancing this variability is $15/MWh [5], although it should be noted that this is not a fully zero-carbon system, requiring the use of gas peakers to manage seasonal variation.

Based on only this information, the conclusion would be that solar and onshore wind are the most cost competitive technologies and fusion would need to compete directly on price at a $50/MWh level. This, however, does not account for an important aspect, which is the balance of supply and demand. Electricity is expected to increase substantially as a fraction of total energy use as sectors currently using hydrocarbons electrify [6,7]. The electrification of transport is an example. Without a significant increase in the rate of deployment of renewables, it seems likely that there will be a ‘clean power gap'. One study estimates the total generation from renewables in the 2030s could reach 50% of demand [8]. This study is global in scope but in relation to the UK, it does not account for the more recent commitment to net-zero, which would imply that the clean power gap is even larger. Fusion must therefore compete with the other options for the remaining market.

The technology choices available to fill that remaining market are dramatically different depending on the weight that is placed on carbon intensiveness, with the choices most constrained in net-zero policy scenarios. If reducing carbon intensity is not of concern, the remaining electricity demand will be met with coal and gas. In a net-zero world, and based only on the economics, this demand would be met with nuclear and the higher cost would be accepted. If nuclear power is not acceptable to the public then the remaining options are limited. Large-scale carbon capture and storage (CCS) is not a proven technology and it roughly doubles the cost of generation, requiring the acceptance of an even higher cost.

Judging what constitutes a competitive price point for fusion is exactly that, a judgement. The author is sceptical about public tolerance for a higher price for electricity and believes that fusion must reach a price point of $100/MWh for the first plants, competing directly with nuclear, and with a pathway for future plants to reach $60/MWh, displacing remaining gas generation. As will be seen, the model presented in this paper suggests that reaching this goal is not only achievable but that inertial fusion may indeed be able to compete directly with renewables on cost.

### (b) Previous studies

There have been several previous conceptual design studies of inertial fusion power plants [9–15]. These prior works focus on singular design points rather than exploration of the parameter space. The works cited include three different types of driver: lasers, heavy ion beams, and pulsed power drivers. Within the laser driver literature there is, over time, a general move in a particular direction, namely, to lower energy drivers and higher frequencies. This in turn leads to lower gains (commensurate with lower driver energies) and a perceived need for very low target costs. The challenges of high frequency are clear, but the benefits are less so, or rather, the reasons for the choice of design point are less clear. Studies of pulsed power drivers focus on lower frequency of operation [15], although this seems to be more out of necessity than design optimization; the replacement of the final transmission line section being a challenging aspect of these designs. The present work aims to complement these prior studies by providing a tool that makes the cost impact of design choices such as higher frequency clearer.

## 2. Model

The model has 14 independent parameters, which are listed in table 2. Using these parameters, the levelized cost of energy (LCOE) is calculated, along with many other outputs such as plant wattage. LCOE is a useful metric through which meaningful comparisons between very different energy generation technologies can be made and is defined as

2.1 $$\text{LCOE}=\frac{\sum_{i=1}^{n}C/{\left(1+d\right)}^{i}}{\sum_{i=1}^{n}E_{i}/{\left(1+d\right)}^{i}},$$

where *C_i_* and *E_i_* are the cost and the energy generated in the *i*th year, respectively, and *d* is the discount rate or weighted average cost of capital. When calculated in this way, the LCOE is the average price for which energy generated would need to be sold for the net present value of the investment to be zero. That is, it is the price that would make the energy generating asset a profitable investment.

**Table 2.** A list of the 14 independent parameters of the model.

parameter	symbol	units
availability	$\mu_{a}$	—
blanket multiple	$E_{b}$	—
discount	d	—
driver cost constant	*γ*	$/J
driver efficiency	$\mu_{d}$	—
driver energy	*E_d_*	J
driver lifetime	*N_d_*	shots/year
frequency	*f*	Hz
gain	G	—
O&M cost constant	*ε*	$/kWe-yr
plant cost constant	*α*	$/kWe
target cost constant	*δ*	$/target
thermal efficiency	$\mu_{th}$	—
yield cost constant	*β*	$/GJ

Typical values for the discount rate depend on many things but a crucial aspect is whether the project is privately funded or government-backed. Privately funded projects might have a discount rate greater than 10%, whereas government borrowing costs can be as low as 2% [16,17].

LCOE is a simplified concept, the price of electricity is not a constant. In many geographies, such as the UK, it is a time-varying quantity set through auction processes in a dynamic market. If a generating asset is being built to address peak, high-price demand, a so-called ‘peaking plant', a more complex calculation of economic viability would be required.

To calculate the LCOE, we need models for cost and energy generated over time. To do this, we will split the plants life into two phases, construction and operation. In this paper, we will take the construction time to be 5 years and the operational lifetime to be 40 years.

The energy generated during construction is zero. The energy generated during operation can be written as

2.2 $$E_{i}=\frac{\left(365\times 24\times 60\times 60\right)}{\left(10^{6}\times 60\times 60\right)}P_{e}\mu_{a},$$

where *P_e_* is the electrical power of the plant, *μ_a_* is the availability, and where the numerical factors convert from power to energy per year in MWh. The availability, normally given as a percentage, is the amount of energy produced compared with continuous operation at rated output. To illustrate by example, for nuclear power availability is mainly set by scheduled downtime. If the refuelling process takes six months and must be done after 3 years, the availability would be 86%.

For a fusion power plant, there is expected to be scheduled downtime for replacement of components, but it should also be expected, particularly for the first plants, that unscheduled downtime will be a major influence on availability. In this analysis, we will take availability as a parameter; a more complex model could have a specific availability sub-model.

The cost model is given by

2.3 $$C_{i}=\{\begin{array}{ll} C_{p}+C_{Y}+C_{d,c\text{on}} & \text{during }\text{construction}\\ C_{t}+C_{O\&M}+C_{d,op} & \text{during }\text{operation} \end{array},$$

where *C_p_* is the plant cost, *C_Y_* is the yield cost, *C_d_*_,con_ and *C_d_*_,*op*_ are the driver costs during construction and operation, respectively, *C_t_* is the target cost and $C_{O\&M}$ is the fixed operations and maintenance cost.

The initial capital expenditure for the construction of the plant is split into three categories: the plant cost, the yield cost and the driver cost. The plant cost is intended to capture the broad balance of plant, including for example the heat exchangers, the steam turbines (if a Rankine cycle is being used), the generator, the connection to grid, the land, the buildings and so on.

It is common practice to describe these costs using $/kWe and in this model we will assume a direct proportionally. This allows us to use proxies from other technologies to provide bounding values. The plant cost is

2.4 $$C_{p}=\frac{\alpha P_{e}}{Y_{c}}$$

where *Y_c_* is the number of years for construction and *α* is a constant describing the plant cost in $/kWe. Nuclear power plants cost around $6000/kWe, whereas gas power plants are closer to $1000/kWe. It is reasonable to assume that the cost of a fusion power plant will be less than the cost of a nuclear plant due to the substantially reduced risk profile, although this should not be taken for granted as generally speaking regulations for fusion do not yet exist. Equally, it is not reasonable to think that a fusion power plant would be less complex to build than a gas power plant, which therefore gives us a lower bound. As a sense check we can put the capital costs from the HYLIFE design [8] into these terms. Separating the driver cost and inflating to 2020 dollars gives $3600/kWe.

Modelling the capital cost as proportional to electrical power only leads to a part of parameter space where costs are minimized using very high fusion yield and low frequency, reducing plant size, and therefore cost, while also reducing target cost. A second aspect of capital cost is therefore added, which is proportional to the fusion energy per event, which here will be called the ‘yield cost'. Essentially this represents the cost of the reaction vessel.

The engineering of a system for large yield but low electrical power, which is the parameter space we're trying to constrain by adding the yield cost, might not be ‘typical'. For example, large energy release and low frequency may lead to a meaningful variation in the temperature of the primary coolant over the shot cycle. This will potentially introduce a complexity in the primary circuit, and possibly a compromise in efficiency. However, to the author, a system with this characteristic does not seem inherently implausible, so long as the central chamber can reliably absorb the energy release.

The yield cost is given by

2.5 $$C_{Y}=\frac{\beta E_{f}}{Y_{c}},$$

where *E_f_* is the fusion energy per shot and *β* is a constant describing the cost in $/GJ. This cost parameter can be bounded by recognizing that the primary coolant itself is, for both the First Light and HYLIFE designs, the most expensive material in the reaction vessel. Both propose pure lithium as the primary coolant. The bulk cost for high-purity lithium metal is roughly $100/kg. All other options, such as FLiBe or enriched lithium, are likely to be more expensive. In comparison, the price of steel is $1–5/kg.

A lower bound for the coolant mass can be found based on the mass required to absorb the fusion energy without egregious temperature rise. Using the heat capacity of lithium and a temperature rise of 400 C gives a cost of $70 k/GJ. An upper bound can be found from the HYLIFE design [8], which has a total lithium inventory of 800 tonnes, giving a cost of $44 M/GJ. It should be noted, however, that the $3600/MWe quoted above includes the lithium cost, i.e. there is a double counting issue with these numbers. This is why the author thinks that the HYLIFE yield cost can be reasonably thought of as an upper bound.

The driver cost appears in both construction and operation because the replacement of parts is an important aspect in the cost of a fusion power plant. The lifetime of the driver itself is an important consideration but there will also be replacement parts required in the reaction vessel and perhaps elsewhere. In this simple model, these costs are all wrapped into a ‘replacement driver' cost, representing a requirement for continued capital expenditure.

The driver cost is often discussed in terms of the cost per Joule of energy delivered to target. In this model, we will use the cost per Joule of energy in the bank. This allows the driver efficiency to be separated from the cost more cleanly and reduces the range of values. The driver cost is given by

2.6 $$C_{d,con}=\frac{\gamma E_{d}}{Y_{c}}$$

and

2.7 $$C_{d,op}=\frac{\gamma E_{d}}{L_{d}},$$

where *E_d_* is the driver bank energy, *L_d_* is the driver lifetime in years and *γ* is a constant describing the driver cost in $/J. Some rough values for *γ* can be found by looking at existing machines. The NIF laser costs roughly $4bn and has a 422 MJ capacitor bank, giving a cost of $9.5/J. First Light's Machine Three has a bank energy of 2.5 MJ and cost $4.3 M, giving a cost of $1.7/J. It should be noted that neither of these are rep rated machines. The increased engineering challenges inherent in a rep rated machine, for example increased wear on components, would likely increase the cost further. A more complex driver cost model could include component level costs and lifetimes specific to the technology being considered.

The driver lifetime in years is given by

2.8 $$L_{d}=\frac{N_{d}}{N_{y}},$$

where *N_d_* is the lifetime of the driver expressed as a number of shots, and *N_y_* is the number of shots per year. Expressing the driver lifetime in terms of number of shots allows an important trade-off with frequency to be captured. The number of shots per year is

2.9 $$N_{y}=(365\times 24\times 60\times 60)f\mu_{a},$$

where *f* is the frequency of shots and where the availability also appears to account for downtime.

The target cost is given by

2.10 $$C_{t}=\delta N_{y},$$

where *δ* is a constant describing the target cost in $/target. It should be noted that ‘target’ cost in this context does not strictly mean the fusion target itself. All components that are consumed in a shot must be accounted for, including, for example, the final transmission line section for the Z-IFE approach [12].

Present inertial fusion targets are extremely expensive, but these are essentially bespoke, one-off prototypes. Ultimately, in a mass manufacturing context, the target cost might reasonably be expected to be proportional to the cost of the raw materials. A more complex model could therefore have a schedule of target materials and a cost per kg for each, possibly with a multiplier accounting for the cost of the manufacturing process itself. In this study, we will simply treat *δ* as a parameter with the aim of finding the requirements for target cost independent of any specific design.

The operations and maintenance cost represents other general running costs, such as staff. These costs are commonly described in terms of $/kWe-yr. Again, figures from gas and nuclear can give us bounds, in this case $10–100/kWe-yr [18].

2.11 $$C_{O\&M}=\varepsilon P_{e},$$

where ε is O&M cost in $/kWe-yr.

This leaves us with only the electrical power, *P_e_*, unaccounted for. This can be expressed as,

2.12 $$P_{e}=\mu_{th}P_{th}-P_{rc},$$

where *μ_th_* is the thermodynamic efficiency, *P_th_* is the thermal power and *P_rc_* is the recirculating power required to run the plant. The main contributor to this will be the driver, therefore,

2.13 $$P_{rc}=2E_{d}f,$$

where an arbitrary pre-factor of two has been applied, notionally motivated by the cooling requirements being equal to the driver power itself. This is another area where a more complex model could be developed. As examples, plants with a liquid metal first wall may have significant pumping power, and plant concepts with replaceable transmission lines may need to account for the energy cost of reforming the transmission lines for each shot.

The thermal power itself, *P_th_*, can be written as

2.14 $$P_{th}=E_{b}P_{\text{fus}},$$

where *E_b_* is the energy multiple in the blanket or primary coolant and *P*_fus_ is the fusion power. The energy multiple comes from the reaction between the fusion neutrons and lithium, which regardless of any energy multiple must be used to produce tritium. The reaction with ^6^Li is exothermic, but the reaction with ^7^Li is endothermic. This means that the overall sum can be endo- or exothermic depending on the approach used. A reasonable range for this value is between 0.6 and 1.4.

Finally, the fusion power is given by

2.15 $$P_{fus}=GE_{t}f,$$

where *G* is the target gain and *E_t_* is the target energy, or more specifically the energy incident on the target, which is given by

2.16 $$E_{t}=\mu_{d}E_{d},$$

where *μ_d_* is the driver efficiency. Describing the model in these terms allows addition of gain curves specific to individual target design concepts. Later we will add two different gain curves and assess the impact on the available parameter space for power plant design. The first is the spherical, isobaric hot spot limiting gain curve [1]

2.17 $$G=6000\mu_{c}{\left(\frac{\mu_{c}E_{d}}{A}\right)}^{0.3},$$

where *μ_c_* = *E_fuel_*/*E_t_* is the coupling efficiency (where *E_fuel_* is the final energy in the fuel), and *A* is the isentrope parameter. In this equation, the driver energy, *E_d_*, is in MJ. This model considers ignition of preassembled fuel and finds there to be an absolute maximum gain as a function of driver energy. It does not consider how the fuel is assembled, which motivates the addition of a second gain curve [19]

2.18 $$\begin{array}{rl} \rho r & ={\left(\frac{3}{4\pi }\frac{\rho^{3}}{e}\mu_{c}E_{d}\right)}^{1/3}=0.178\times E_{d}^{1/3}, \end{array}$$

2.19 $$\begin{array}{rl} \theta & =\frac{\rho r}{\rho r+70} \end{array}$$

2.20 $$\begin{array}{rl} \;\text{and}\;G & =6.75e14\times \frac{\theta \mu_{c}}{V^{2}}, \end{array}$$

where *ρr* is the fuel areal density, *ρ* is the fuel mass density, *e* is the fuel internal energy density, *θ* is the burn fraction and *V* is the implosion velocity. The scaling of areal density with driver energy had to be reconstructed from the values given in the reference. Essentially this means finding the value for *ρ*^3^/*e*. This has been done using the hydrodynamic equivalence concept discussed in the reference. That is, the implosion velocity, final fuel internal energy density and final fuel mass density were all assumed to remain constant as the driver energy increases. That is, the fuel mass increases but all intrinsic properties remain the same. This process was followed for a coupling efficiency of 10% and an implosion velocity of 400 km s^−1^, meaning that the numerical factor in equation (2.18) is only valid for these values.

In adding these gain curves to the model, we have removed gain as a free parameter but added further new parameters describing fundamental aspects of target physics. We will initially work with the gain as a free parameter with the aim, much like target cost, of understanding what is required without reference to any specific approach.

## 3. Results and discussion

### (a) Correlation analysis

To explore the properties of the model a Monte Carlo approach was taken. There are 14 parameters of interest, which are listed in table 3. The table also shows the ranges used for each of the individual values, which were sampled uniformly in linear or log space as indicated. Ten million samples were used. Some combinations give rise to designs that are clearly unfeasible, for example, having extremely high fusion yield. The results were filtered such that the electrical power is less than 2 GWe, the LCOE is less than $200/MWh, the initial capital expenditure is less than $10bn, and the fusion energy is less than 25 GJ.

**Table 3.** A list of the parameters in the model, the ranges used for a Monte Carlo sample over all parameters, whether the particular parameter is sampled uniformly in linear or log space, and the resulting Pearson correlation coefficient between the individual parameter of interest and the LCOE.

input parameter	range	linear or log space	Pearson correlation coefficient
discount	2–12%	linear	+0.247
plant cost	$1000–6000/kWe	linear	+0.210
target cost	$1–100/target	log	+0.186
gain	10–1000	log	−0.164
driver lifetime	10^6^–10^9^ shots	log	−0.134
availability	50–100%	linear	−0.127
driver cost	$2–10/J	linear	+0.075
driver efficiency	5–30%	linear	−0.063
O&M cost	$10–100/kWe-yr	linear	+0.050
blanket multiple	0.6–1.4	linear	−0.038
thermal efficiency	30–60%	linear	−0.033
frequency	0.01–10 Hz	log	+0.035
yield cost	$500 k–50 M/GJ	log	+0.026
target energy	0.5–50 MJ	linear	+0.011

To compare the relative importance of the different parameters the Pearson correlation coefficient was calculated between each of the input parameters individually and the LCOE. The Pearson correlation coefficient is given by

3.1 $$\rho =\frac{cov(A,B)}{\sigma_{A}\sigma_{B}}$$

where *cov*(*A*, *B*) is the covariance between variables *A* and *B*, and *σ_A_* and *σ_B_* are the standard deviations of *A* and *B*, respectively. The value of the correlation coefficient is between −1 and +1, indicating perfect negative and positive correlations, respectively.

Values for the Pearson correlation are given in the final column of table 3. The results do not indicate a dominant correlation with any parameter, but there are some that have a larger impact than others. A purely financial aspect, the discount rate, has the strongest influence on LCOE. This should not come as a surprise for the reader familiar with costs in the nuclear sector, where the difference between China and Europe, for example, is in large part explained by the different cost of capital.

The plant cost features next. The plant cost depends on the engineering of the wider balance of plant. Its importance underlines the importance of a regulatory regime appropriate to the specific risks and challenges of fusion.

The next two aspects relate to the target, the target cost and the gain. The results show a greater correlation between target cost and LCOE than gain, which suggests it is more important to have a cheaper target than higher gain. The difference between the coefficients is not large, however.

After these is a grouping of four parameters relating to the driver, with the driver lifetime and availability stronger influences than the raw driver cost itself. This implies that reliable operation is the most important aspect for the driver.

The remaining parameters do not have a strong influence. The most surprising finding here might be the very weak influence of yield cost. This does, however, agree with more detailed proprietary studies conducted by First Light, which found a weak dependence between details of the reaction vessel and LCOE.

The weak dependence on frequency appears to contradict some more detailed studies, such as HiPER, which proposes 10 Hz operation [14]. This needs deeper investigation and shows the limits of the analysis here; in some cases, it is not enough to understand the global dependence. In the following section, individual dependences will be explored but before moving on, we can also look at correlations between LCOE and other output variables.

The model shows an ‘economy of scale', with larger plants found to correlate with lower LCOE. The Pearson correlation coefficient between electrical power and LCOE is −0.233, the second-strongest correlation found. This needs exploration because the plant cost is linear with electrical power. To explain this dependence, we need to look at the recirculating power as a fraction of the total. The correlation between this and LCOE is +0.171, which confirms the instinct that the recirculating power must be a small fraction of the total.

### (b) Dependence on individual parameters

In the following, unless otherwise stated, the default values are: availability = 70%; driver lifetime = 50 M shots, which at 0.2 Hz is about 8 years; thermal efficiency = 40%; discount rate = 8%; plant cost = $3 k/kWe; driver cost = $5/J; target cost = $10/target, yield cost = $5 M/GJ; O&M cost = $30/kWe-yr; target energy = 10 MJ; frequency = 0.2 Hz; gain = 500; driver efficiency = 10%; and blanket multiple = 1.2.

First, we will examine the parameters relating to cost. Detailed analysis of the discount, plant cost and driver cost did not reveal anything particularly surprising other than confirming expectations from the generally weak correlation with any individual parameter. Even if one of these values is at the top of the range there are still design points with LCOE less than $80/MWh achievable.

**Figure 1.**
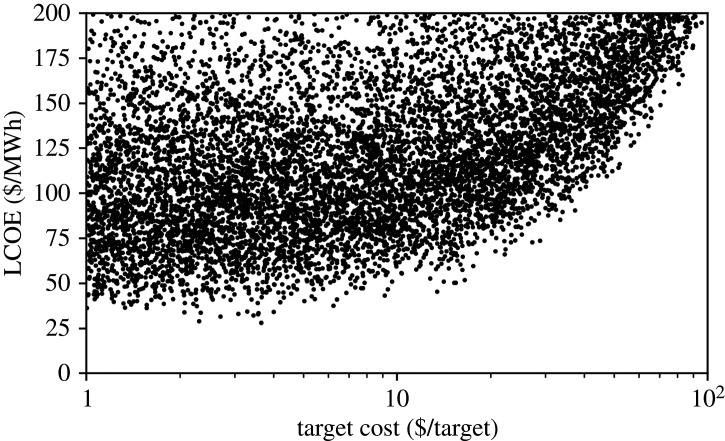
LCOE versus target cost. All six cost parameters are sampled over the ranges indicated in table 3, with 10 k samples used. The results show that once target cost is less than roughly $10 the impact of further cost reduction is not large.

Figure 1 shows the dependence of LCOE on target cost. There is clearly a nonlinear relationship where, essentially, if the target cost is large it begins to dominate the costs overall. Broadly speaking it seems that there is a threshold below which the target cost has been reduced enough and after which there is limited benefit for further improvement, and that that threshold is roughly $10/target.

Exploration of the yield cost did not reveal anything to change the conclusion from the general correlation analysis, which is that it does not play a large role. To put this in context, for the default design point here, the fusion yield is 5 GJ per event. At the upper end of the range, this means that the reaction vessel alone costs $250 M. However, the plant cost is $1.3bn and the driver cost is $500 M in this case, which explains the insensitivity.

Moving onto parameters relating more to engineering and physics, the dependence on driver lifetime is as expected, with a longer lifespan naturally being better. It is interesting to note, however, that like target cost, there seems to be a threshold beyond which further improvement delivers marginal benefit. For the default parameters here this is about 5 years, which is roughly 30 M shots.

**Figure 2.**
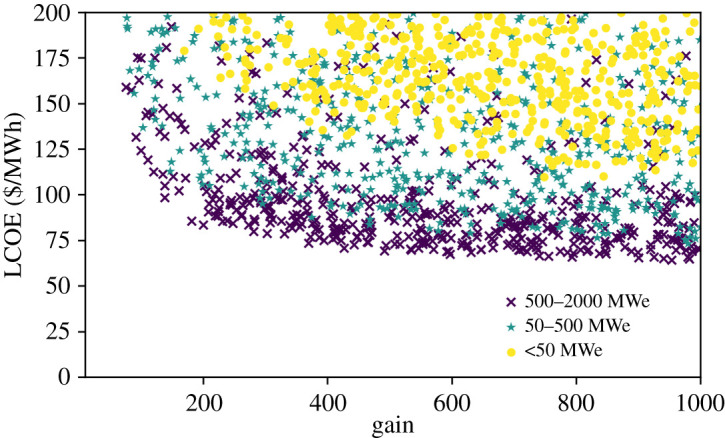
LCOE versus gain. Gain, driver energy, frequency and driver efficiency are being sampled over the ranges indicated in table 3. The points are coloured by electrical power as indicated in the legend. In order to fill out the less common parameter space, which are the smaller plant sizes, 100 k samples were required. The results were truncated to show 500 points per category. The dependence of LCOE on both gain and electrical power can be seen. (Online version in colour.)

LCOE as a function of gain is shown in figure 2, with a stratification by electrical power. This shows that the gain needs to be above a certain level to reach a cost competitive design; for the default design here, greater than roughly 400. The required gain depends on the cost parameters, however. If values near the bottom of the range are accessible, competitive LCOE numbers exist even with gain less than 100.

The figure also shows two things about plant size. First, that smaller plants require higher gain, which might seem counterintuitive. Second, the dependence of LCOE on the plant size can be seen, although a wide range of values had to be selected to make the effect clearly visible. Perhaps the most surprising conclusion from this plot is that designs smaller than 50 MWe can potentially be viable.

**Figure 3.**
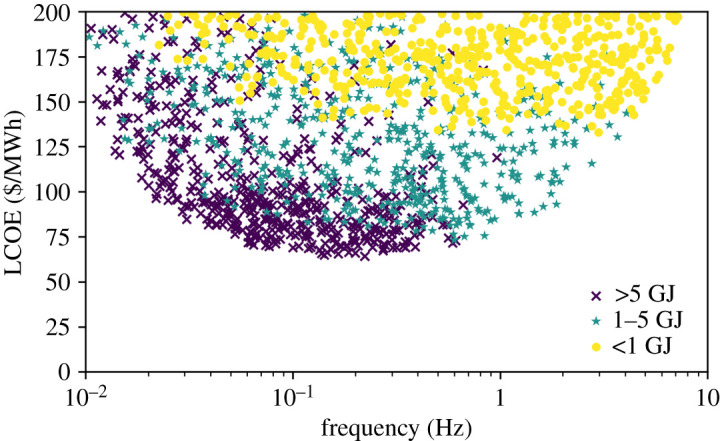
LCOE versus frequency, coloured by fusion energy per shot. The sampling is as in figure 2, with the low yield designs the less common in this case. An optimum frequency can be seen, but the location of this depends on the fusion yield, with lower yield designs having an optimum at higher frequency. (Online version in colour.)

Figure 3 shows LCOE versus frequency, where the results have been stratified according to the fusion energy per shot. With no restriction on yield, there is an optimum frequency. On the left, the driver and/or reaction vessel increase in cost. On the right the limit on electrical power, in this case 2 GWe, clips the parameter space. If the yield is constant and the frequency increases, the power must increase. With no limit on power output there is no optimum frequency, with faster being universally better. Returning to the correlation analysis without the electrical power and capital expense limits confirms this, with the new correlation coefficient being −0.110.

Looking at the different yield categories seems to explain why existing ICF designs have high electrical power and high frequency. As the yield decreases, the optimum frequency increases, and with a yield of 1 GJ or less the optimum is at the cut-off for electrical power. This suggests that the energy per event has a profound, although indirect, influence on the optimal design point for ICF. Higher yield enables lower LCOE through reduced target cost and reduced plant cost, while high gain ensures that recirculating power is minimized.

### (c) Addition of gain curves

The first gain curve [1], the limiting gain curve for a spherical, isobaric hot spot fuel configuration, given in equation (2.17), replaces gain as an independent parameter with two new physics parameters: *μ_c_*, the coupling efficiency, and *A*, the isentrope parameter. These were set to 10% and 3, respectively. In the Monte Carlo approach used, the gain was randomly set to a value between 1 and the limiting gain, where that limiting gain was commensurate with the randomly selected driver energy used for that calculation. The results showed little change, indicating that this fuel configuration is fundamentally able to deliver enough gain to reach cost competitiveness.

The second gain curve [19], given in equations (2.18–2.20), is much more restrictive in terms of absolute gain attainable, reaching a maximum of approximately 250 for the driver energies considered here. This gain curve adds the fuel mass density, *ρ*, the fuel internal energy, *e*, the implosion velocity, *V*, and the coupling efficiency, *μ_c_*, as independent parameters. The same approach to sampling is used as for the first gain curve, i.e. between a gain of one and the value specified by the gain curve.

**Figure 4.**
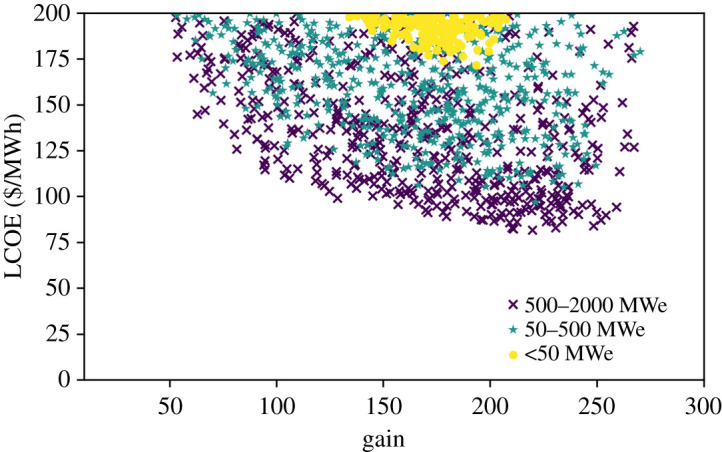
LCOE versus gain, where the gain is not an independent parameter but has been calculated using a gain curve from [19]. Driver energy, frequency and driver efficiency are being sampled over the ranges indicated in table 3. Note that the *x*-axis is truncated at a gain of 300. With this gain curve there is a cost optimum very close to the ultimate gain limit specified by the gain curve. 500 k samples were used. (Online version in colour.)

The results are shown in figure 4. In this case the impact is more pronounced; the limit on gain removes the lowest cost designs, which need gains greater than approximately 400. The smaller plant sizes are affected more than the larger ones by this restriction. The degree of compromise also depends on the actual cost levels, which in figure 4 remain at the defaults. Broader exploration of the results shows low-cost design points remain even with the more stringent limit on gain.

### (d) Minimum cost design point

**Figure 5.**
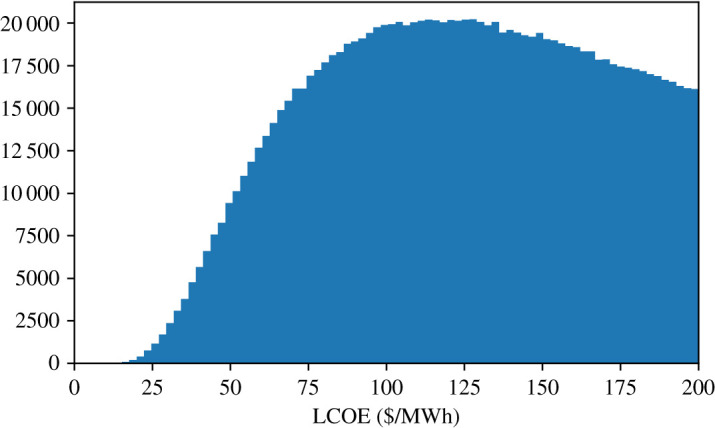
A histogram of the LCOE from the sample over all parameters. The scan shows that very low-cost designs, less than $25/MWh, may be possible.

We will now return to the scan over all parameters but with the intention of understanding the lowest cost design points. Figure 5 shows a histogram of all calculated values for LCOE and appears to show the possibility of very low-cost configurations.

An example configuration for which the model finds an LCOE of $24.6/MWh is: availability = 80%; driver lifetime = 40 M shots; thermal efficiency = 50%; discount rate = 4%; plant cost = $1.5 k/kWe; driver cost = $3/J; target cost = $2/target, yield cost = $5 M/GJ; O&M cost = $20/kWe-yr; target energy = 5 MJ; frequency = 0.05 Hz; gain = 1000 and driver efficiency = 20%. This results in a thermal power of 300 MWth and electrical power 147.5 MWe.

The dominant cost is the initial capital expenditure, where the plant cost is $220 M, the driver cost is $75 M, and the yield cost is $25 M. The driver cost is low because of the low target energy and high efficiency. Whether a system able to meet the frequency and lifetime requirements could really be built for this cost is very uncertain.

The other question is about the plant costs. Costs for combined-cycle gas power plants, where a Rankine cycle and the associated space and infrastructure is required, can be less than $1000/kWe; however, this number is based on a 700 MWe plant [14]. From a physics point of view, gain of 1000 is very high. The limiting gain curve of Atzeni and Meyer-ter-Vehn [1] shows it is possible to reach this level, but gain of 1000 is certainly larger than typically discussed.

Clearly, design points of this type are worth further exploration, but the author urges caution. It is the role of a simple model to indicate interesting parts of parameter space, but much more detailed study is required to substantiate the findings.

## 4. Conclusion

The aim of this work was to build a simple model for LCOE of an inertial fusion power plant. The core physics has been described in a technology agnostic manner that allows broad application across different inertial fusion approaches. The model for the wider system has been built to maximize the use of proxies from existing technology, allowing a comparative approach. The model seems to capture important trade-offs in the design and allows cost competitive design points to be rapidly identified and explored.

A Monte Carlo approach and correlation analysis revealed that no single parameter is strongly dominant. The discount rate, plant cost and target cost were found to be the most important aspects globally, but detailed analysis revealed complex trends in some areas, more so than can be captured in a single correlation coefficient.

One such area was the target cost, which was found to decrease in importance below a cost threshold of roughly $10/target. Another is the required gain. This work opened with the oft-cited requirement for a gain greater than 30–100 for power production for ICF [1]. The present model extends this analysis to include the question of cost competitiveness and finds that LCOE always benefits from higher gain. There is, however, a rough threshold beyond which further increases in gain only delivers marginal benefit. For the mid-range default parameters used in this study, this gain threshold is around 400. It should be noted, though, that constraining gain to a maximum of approximately 250 still led to competitive designs, although the lowest cost points were closed off.

Perhaps the most complex aspect identified was in the analysis of LCOE versus frequency. This highlighted a complex relationship between optimal frequency and the fusion energy per shot and illustrates what is, in the author's mind, the most important conclusion of this work. As the amount of energy per shot increases, the optimum frequency decreases. Not only does this reduce one of the main engineering challenges of inertial fusion, it also appears to unlock a fundamentally lower LCOE. Moreover, this type of design point does not seem to be confined to very large plant sizes. A potential design was identified at approximately 150 MWe scale. In this case, the engineering challenges of the wider plant are reduced due to the small size, the engineering challenges in the centre of the plant are reduced by the low frequency, the financing challenge is reduced again through the small size and commensurate lower initial capital cost, and the LCOE is also very attractive.

There are of course compromises. The engineering challenge of designing a reaction vessel to accept multi-GJ yields must be solved, and the physics challenge of high gain must also be addressed. The design point also relies on a cheap and efficient driver design.

Regarding future study, the properties of the present model have by no means been exhaustively explored. It is also easily extensible through the addition of more detailed sub-models, for example a more detailed cost and lifetime model of a prospective driver technology, or gain curves capturing relevant physics in a more detailed manner than those included here. The model would also be able to facilitate like-for-like comparison of different approaches by providing a common basis.

To conclude, there remains much to be understood about the ultimate potential of inertial fusion to deliver cost competitive electricity, but the results presented here show that it has the potential to be a very attractive option.

## Supplementary Material

Source code of the model described in the paper

## Data accessibility

The source code for the work is included in the electronic supplementary material.
